# Downregulation of hsa_circ_0005243 induces trophoblast cell dysfunction and inflammation via the β-catenin and NF-κB pathways

**DOI:** 10.1186/s12958-020-00612-0

**Published:** 2020-05-20

**Authors:** Huiyan Wang, Wenbo Zhou, Guangtong She, Bin Yu, Lizhou Sun

**Affiliations:** 1 Department of Obstetrics, The First Affiliated Hospital of Nanjing Medical University, Nanjing, 210000 Jiangsu China; 2 Department of Obstetrics, Changzhou Maternity and Child Health Care Hospital affiliated Hospital Affiliated to Nanjing Medical University, Changzhou, 213003 Jiangsu China

**Keywords:** β-Catenin, circRNA, Gestational diabetes mellitus, hsa_circ_0005243, NF-κB

## Abstract

**Background:**

Gestational diabetes mellitus (GDM) is a common complication in pregnancy that poses a serious threat to the health of both mother and child. While the specific etiology and pathogenesis of this disease are not fully understood, it is thought to arise due to a combination of insulin resistance, inflammation, and genetic factors. Circular RNAs (circRNAs) are a special kind of non-coding RNA that have attracted significant attention in recent years due to their diverse activities, including a potential regulatory role in pregnancy-related diseases, such as GDM.

**Methods:**

We previously reported the existence of a novel circRNA, hsa_circ_0005243, which was identified by RNA sequencing. In this study, we examined its expression in 20 pregnant women with GDM and 20 normal pregnant controls using quantitative reverse transcription PCR analysis. Subsequent in vitro experiments were conducted following hsa_circ_0005243 knockdown in HTR-8/SVneo cells to examine the role of hsa_circ_0005243 in cell proliferation and migration, as well as the secretion of inflammatory factors such as tumor necrosis factor alpha (TNF-α) and interleukin 6 (IL-6). Finally, we examined the expression of β-catenin and nuclear factor kappa-B (NF-κB) signaling pathways to assess their role in GDM pathogenesis.

**Results:**

Expression of hsa_circ_0005243 was significantly reduced in both the placenta and plasma of GDM patients. Knockdown of hsa_circ_0005243 in trophoblast cells significantly suppressed cell proliferation and migration ability. In addition, increased secretion of inflammatory factors (TNF-α and IL-6) was observed after hsa_circ_0005243 depletion. Further analyses showed that knockdown of hsa_circ_0005243 reduced the expression of β-catenin and increased nuclear NF-κB p65 nuclear translocation.

**Conclusions:**

Downregulation of hsa_circ_0005243 may be associated with the pathogenesis of GDM via the regulation of β-catenin and NF-κB signal pathways, suggesting a new potential therapeutic target for GDM.

## Introduction

Gestational diabetes mellitus (GDM) is a form of diabetes characterized by glucose intolerance and insulin resistance beginning or first recognized during pregnancy [1, 2]. GDM may affect as many as 17.5% of all pregnant women in China [3], resulting in a significantly increased risk of developing metabolic syndrome and type 2 diabetes after delivery [4]. Therefore, timely diagnosis and appropriate therapeutic intervention are important for reducing the risk of adverse pregnancy outcomes in GDM patients.

While the specific etiology and pathogenesis of GDM are not fully understood, the disease is thought to arise as a result of insulin resistance, inflammation, dysfunction of islet beta cells, and genetic factors. The placenta is a highly specialized organ that serves as the interface between maternal and fetal circulation during pregnancy. The mechanisms of placental pathology during diabetes are still largely unclear. However, research has demonstrated a significant inflammatory response in the placental tissues of GDM, characterized by an increase in the number of macrophages and the content of saturated fatty acids, leading to the release of inflammatory factors interleukin IL-6, IL-8, and toll-like receptor 2 by trophoblast cells [5].

Circular RNAs (circRNAs) are a form of RNA consisting of a closed loop, and have attracted major attention in recent years [6, 7]. With the development of high-throughput sequencing technology, increasing numbers of circRNAs have been discovered, revealing a wide array of biological characteristics and regulatory functions. circRNAs are highly conserved across species in terms of stability, diversity, and tissue specificity [6, 8]. They participate in the regulation of gene expression at both the transcriptional and post-transcriptional levels, through which they have been shown to play roles in numerous physiological and pathological processes [9, 10]. Emerging evidence has shown that circRNAs are strongly associated with the occurrence and development of preeclampsia, GDM, and other pregnancy-related diseases [11, 12]. Previously, we reported a significant decrease of hsa_circ_0005243 in the placenta of GDM pregnant women by high-throughput RNA sequencing [13]. Here, we investigated the possible regulatory function of this circRNA in the trophoblast cell line HTR-8/SVneo and explored its potential mechanisms of action.

## Materials and methods

### Patients

From April 2017 to December 2018, we identified 20 parturient women diagnosed with GDM and 20 parturient healthy control women in the obstetrics department of the Changzhou Maternal and Child Health Care Hospital, an affiliated hospital of Nanjing Medical University. Placenta tissues were obtained within 10 min after delivery, while the maternal plasma specimens were collected on an empty stomach early in the morning on the day of hospitalization during the third trimester (37–40 weeks). After collection, the plasma and placenta samples were stored at − 80 °C. All GDM and control women were matched by age and body mass index. The diagnosis of GDM was conducted by 75-g oral glucose tolerance test at 24–28 weeks. Exclusion criteria included multiple births, premature delivery, delivery age < 20 years old or > 40 years old, diabetes, chronic liver and kidney diseases, thyroid and other endocrine diseases, and hypertension prior to pregnancy. The clinical characteristics of the enrolled pregnant women are listed in Table 1. Informed consent was obtained from each participant and this study was approved by the ethics committee of the hospital.

**Table 1:** Clinical parameters of GDM and normal controls

Characteristic	GDM(*n* = 20)	Control(*n* = 20)	*p* value
Age (year)	32.26 ± 4.06	30.4 ± 4.35	0.176
Gestational age (day)	38.63 ± 0.59	38.81 ± 0.71	0.403
Pre-pregnancy BMI index (kg/m2)	22.17 ± 2.1	21.04 ± 1.95	0.082
Late-pregnancy BMI index (kg/m2)	27.74 ± 1.73	26.39 ± 2.07	0.032
OGTT 0 h	5.32 ± 1.58	4.4 ± 0.36	0.021
OGTT 1 h	10.35 ± 2.95	7.37 ± 1.33	0.001
OGTT 2 h	9.24 ± 3.73	6.24 ± 1.05	0.003
HbA1c(%)	5.37 ± 0.9	4.9 ± 0.37	0.051
Birth weight(g)	3529 ± 527.2	3306 ± 379.64	0.143

*BMI* body mass index. Values were expressed as means ± standard deviation

### Cell culture and transfection

Human trophoblast HTR-8/SVneo cells were cultured in 1640 medium supplemented with 10% fetal bovine serum (FBS; Gibco) and 1% penicillin/streptomycin at 37 °C with 5% CO_2_. Cells (2 × 10^5^) were seeded in 6-well plates and transfected with small interfering RNAs (siRNAs) targeting hsa_circ_0005243 using Lipofectamine 3000 (Invitrogen, USA) according to the manufacturer’s protocol. The knockdown efficiency of siRNAs was determined by quantitative reverse transcription (qRT) PCR using the following primers: siRNA-1, 5′-UGA CCA UCA UCU ACA ACA UTT-3′, 5′-AUG UUG UAG AUG AUG GUC ATT-3′; siRNA-2, 5′-CCA UGA ACC CGC ACG ACA UTT-3′, 5′-AUG UCG UGC GGG UUC AUG GTT-3′; siRNA-3, 5′-CCU ACA AGG UCU AUG CUG ATT-3′, 5′-UCA GCA UAG ACC UUG UAG GTT-3′. All siRNAs and negative controls (NCs) were obtained from RiboBio (Guangzhou, China).

### CCK8 assay

Cells were trypsinized and seeded into 96-well cell culture plates (Corning Inc., Corning, NY, USA) at a concentration of 3 × 10^3^ cells/mL. Cell viability was measured after culture for 24, 48, and 72 h by adding 10 μL of CCK8 reagent (DOJINDO Laboratories, Kumamoto, Japan). Cells were then incubated at 37 °C for 3 h, after which the optical density at 450 nm (OD_450_) was assessed using a microplate reader (BioTek, Winooski, VT, USA).

### Colony formation assay

Cells in logarithmic phase growth were trypsinized, re-suspended, and inoculated in 6-well cell culture plates containing 2 mL of medium. After plating, the culture plates were gently shaken to ensure even distribution of the cells within the wells and placed in an incubator at 37 °C and 5% CO_2_ for 24 h until full adherence was obtained. After 12 days, the medium was discarded, and the cells were carefully soaked twice with phosphate-buffered saline (PBS). Cells were then fixed for 15 min with 5 mL of absolute ethanol. After discarding the fixative solution, the cells were treated with Giemsa dye solution (Thermo Fisher Scientific, Waltham, MA, USA) for 10–30 min, followed by slow washing with running water. Finally, cells were air dried, photographed, and counted.

### EdU assay

To evaluate the proliferation ability of trophoblast cells, an EdU assay was performed using a keyFluor 555 Click-iT EdU imaging detection kit (Keygen Tec, Nanjing, China) according to the manufacturer’s protocol. Briefly, cells were fixed with 4% paraformaldehyde, then incubated with 2 mg/mL glycine for 5 min, followed by 200 μL of 1× Apollo staining solution for 30 min in a bleached shaker at room temperature, away from light. Cells were then washed with PBS, after which 100 μL of penetrant agent (0.5% Triton X-100 in PBS) was added. Cell nuclei were stained with Hoechst 33342, and the cells were photographed with a high-content imaging system (MD Micro Solutions, Gloucester, MA, USA).

### Migration assay

For the in vitro transwell migration assay, the transfected cells were trypsinized, adjusted to a density of 1 × 10^5^ cells/mL, and 100 μL of cell suspension and 700 μL of medium containing FBS were added to the upper and lower chambers of a transwell plate (Corning Inc.). The cell culture plates were then placed in an incubator at 37 °C with 5% CO_2_ for 24 h. Cells in the upper chamber were removed using a cotton swab, while the cells on the lower surface of membranes were fixed with formaldehyde and stained with 0.1% crystal violet (Sigma-Aldrich, St. Louis, MO, USA). After incubation at 37 °C for 30 min, cells were washed with PBS, and three to five fields were randomly selected and photographed, with the number of migrated cells counted under an inverted microscope (Olympus, Tokyo, Japan).

For the wound-healing assay, cells in logarithmic growth phase were trypsinized and inoculated into a 6-well plate. After 24 h, when the cell aggregation reached ~ 60%, a sterile nozzle was used to evenly draw lines in the plate. Floating cells were removed by washing with PBS, and then fresh medium added for further culture. After 24 h, the cells were taken out and photographed (200× magnification), and the migration distance of cells was measured.

### Enzyme-linked immunosorbent assay

Cells were seeded in 6-well plates (Corning Inc.) and transfected as described above, after which the medium was collected and replaced with fresh culture medium. The culture medium was then centrifuged for 20 min at 1000×*g* to remove cell debris and impurities. The concentrations of tumor necrosis factor alpha (TNF-α) and IL-6 in the medium were detected using a commercially available enzyme-linked immunosorbent assay (ELISA) kit (Mlbio, Shanghai, China) according to the manufacturer’s protocol. The absorbance (OD_450)_ of each group was measured using a microplate reader (MD SpectraMax M3; Molecular Devices, San Jose, CA, USA).

### Western blot

Transfected cells were harvested and lysed in lysate buffer containing protease inhibitors. Protein concentration was determined using a BCA kit (Thermo Fisher Scientific). After denaturation, the proteins were separated using 12% sodium dodecyl sulfate polyacrylamide gel electrophoresis, and then transferred to a polyvinylidene fluoride membrane (Merck Millipore, Darmstadt, Germany) and blocked with 5% skim milk. Membranes were then incubated in the presence of primary antibodies at 4 °C overnight at the following concentrations: anti-c-myc (1:2000), anti-cyclinD1 (1:3000), anti-survivin (1:3000), anti-β-catenin (1:1000), anti-p65 (1:2000), anti-laminin B (1:3000), and anti-β-actin (1:3000). All antibodies were purchased from Abcam (Cambridge, UK). The membranes were then washed with tris-buffered saline–Tween 20 (TBST) and incubated with horseradish peroxidase-conjugated anti-mouse or anti-rabbit secondary antibodies (Beyotime, China) for 1 h. Membranes were washed again in TBST, incubated with enhanced chemiluminescence reaction reagent (BeyoECL Plus; Beyotime), and visualized using a luminescence imaging system (Tanon, Shanghai, China).

### qRT-PCR

Total RNA was extracted using TRIzol reagent (Thermo Fisher Scientific). The expression of circRNA and *GAPDH* were detected using the SYBR Premix Ex Taq system (Takara, Madison, WI, USA) following the manufacturer’s instructions. RT-PCR was performed using the following primers: hsa_circ_0005243, forward, 5′-TTATCTACATGCACCTGCGCT-3′, reverse, 5′-AAGTGACAAGCTAGCCCTCAT-3′; GAPDH, forward: 5′-CAAATTCCATGGCACCGTCA-3′, reverse: 5′-AGCATCGCCCCACTTGATTT-3′. PCR reactions were conducted as follows: denaturation at 95 °C for 10 min, amplification at 40 cycles of 95 °C for 10 s and 58 °C for 15 s, followed by elongation at 70 °C for 30 s. Relative expression levels were determined by comparing the Ct values of the target genes to those of the *GAPDH* gene.

### Flow cytometry

To assess cell apoptosis, transfected cells were treated with 0.25% trypsin (without EDTA) and collected, washed twice with PBS, stained using an annexin V–FITC apoptosis detection kit (Beyotime), and analyzed by flow cytometry (FACSCalibur; BD, Franklin Lakes, NJ, USA). The number of apoptotic cells was determined by counting and expressed as a ratio relative to live cells.

### Immunofluorescence

Cells were fixed in 4% polyformaldehyde, washed with PBS, and treated with 0.5% Triton X-100, after which the slides were blocked with 5% bovine serum albumin for 30 min. Cells were then incubated with antibodies against β-catenin and p65 (1:100; Abcam) at 4 °C overnight. Next, slides were washed three times in PBS, after which FITC-conjugated secondary antibody (1:100; Abcam) was added and incubated at 37 °C for 1 h in the dark. Finally, slides were stained with DAPI for 5 min, and the expression of protein in cells was observed under a laser confocal microscopy (LSM710; Zeiss, Oberkochen, Germany). Three photographs were randomly taken per slide.

### Statistical analysis

All data were analyzed using SPSS ver. 22 software (IBM Corp., Armonk, NY, USA), with continuous variables expressed as the mean ± standard error. A two-tailed Student’s *t*-test was used to compare the mean of the two sets of samples. The diagnostic value of hsa_circ_0005243 for GDM was established by a ROC curve and the AUC was calculated (adjusted by BMI and age). *P* values < 0.05 were considered statistically significant.

## Results

### Characterization of hsa_circ_0005243 in GDM

**Fig. 1:**
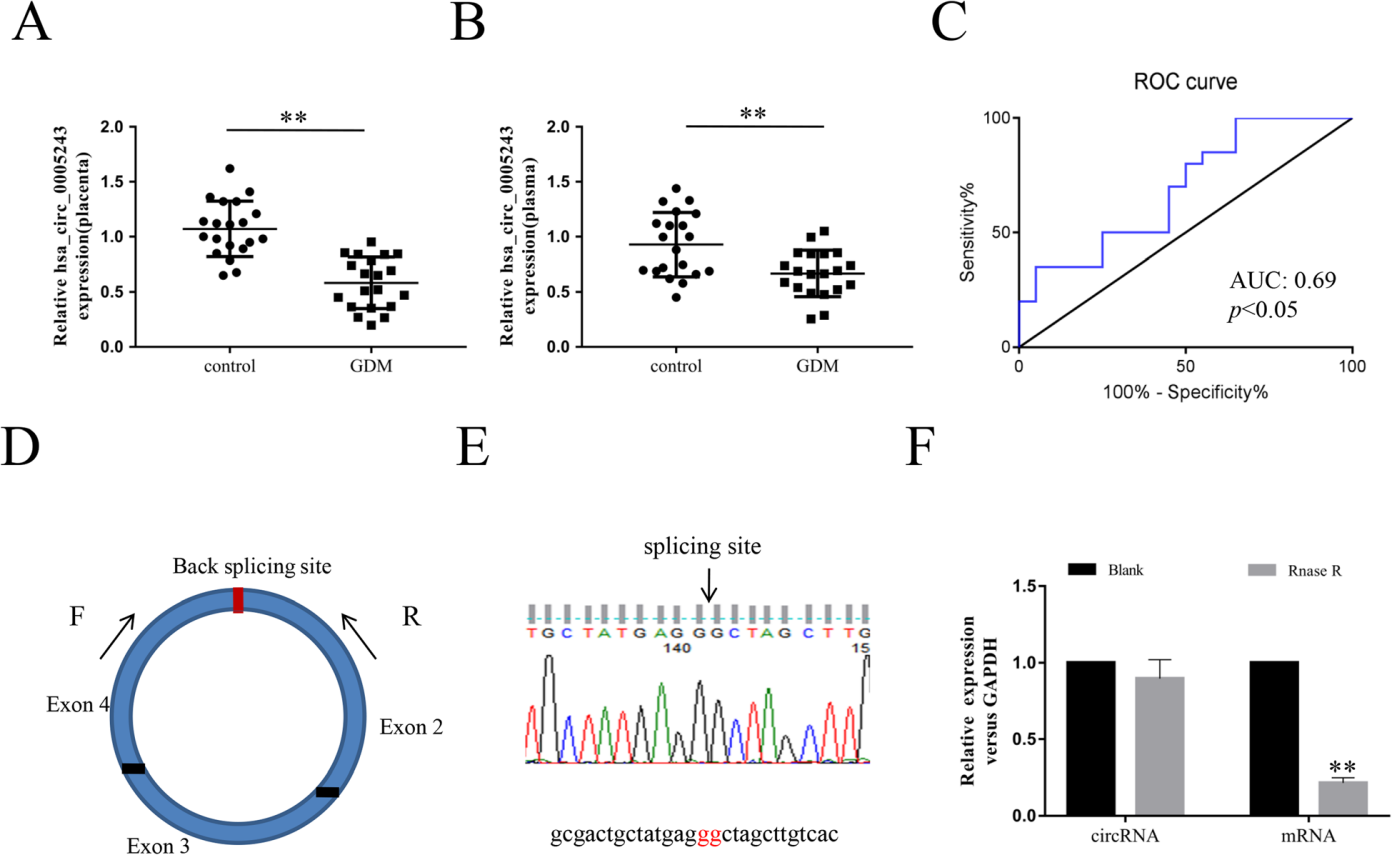
Characterization of hsa_circ_0005243 in GDM. **a**, **b** Relative expression of hsa_circ_0005243 was determined by qRT-PCR in placenta tissues (**a**) and plasma (**b**) of GDM patients and normal controls. **c** ROC curve analysis was used to assess the diagnostic value of hsa_circ_0005243. **d** hsa_circ_0005243 is generated from the back-splicing of exons 2, 3, and 4 of the *TMEM184B* gene. **e** Sanger sequence analysis was used to confirm the splicing site. **f** qRT-PCR analysis of hsa_circ_0005243 and the linear TMEM184B mRNA after treatment with or without RNase R in HTR-8/SVneo cells

Previously, we found that the expression of hsa_circ_0005243 was significantly lower in GDM placenta relative to the control group by high-throughput RNA sequencing [13]. qRT-PCR analyses verified these results, showing that the expression of hsa_circ_0005243 was significantly lower in both the placenta (Fig. 1a) and plasma (Fig. 1b) of GDM patients compared with controls. Based on these findings, the diagnostic value of hsa_circ_0005243 in plasma was further evaluated by receiver operating characteristic (ROC) curve analysis (Fig. 1c), revealing an area under the curve of 0.69 (*p* < 0.05). hsa_circ_0005243 originates from the *TMEM184B* (transmembrane protein 184B) gene and consists of the head-to-tail splicing of exons 2, 3, and 4 with a total length of 517 bp (Fig. 1d). In addition, we found that hsa_circ_0005243 was resistant to RNase R treatment compared with linear mRNA (Fig. 1d).

### Downregulation of hsa_circ_0005243 suppresses trophoblast proliferation and induces apoptosis

**Fig. 2:**
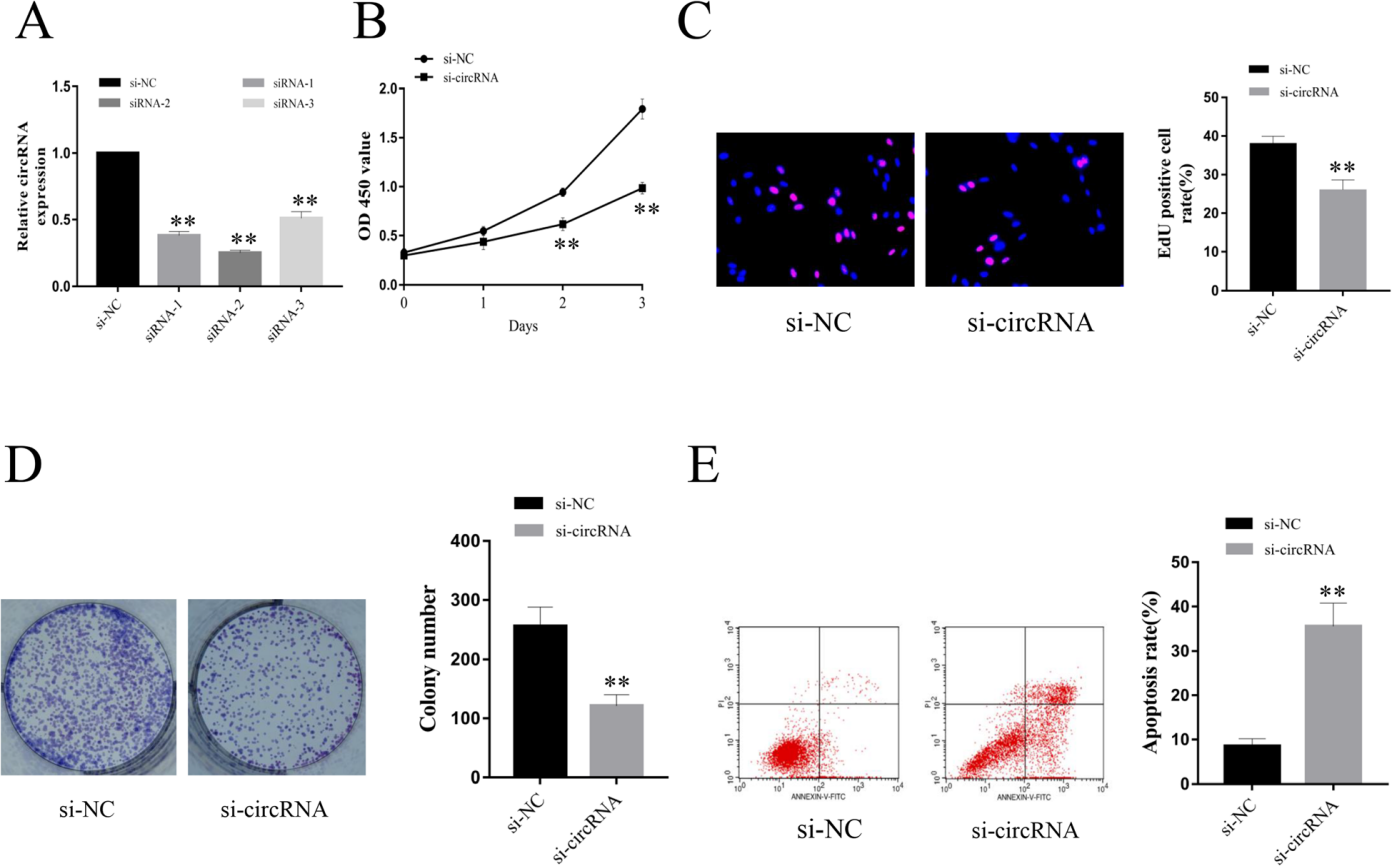
Downregulation of hsa_circ_0005243 suppresses trophoblast cell proliferation and promotes apoptosis. **a** hsa_circ_0005243 expression was determined after transfection with siRNAs. **b** CCK8 analysis revealed a significant reduction in cell viability following hsa_circ_0005243 knockdown. **c**, **d** EdU (**c**) and colony formation (**d**) assays showed that depletion of hsa_circ_0005243 inhibited cell proliferation. **e** Flow cytometry revealed an increase in apoptosis following hsa_circ_0005243 knockdown. Data are presented as the mean ± SD, **p < 0.01

To further explore the potential functions of hsa_circ_0005243 in trophoblast cells, siRNAs (si-circRNA and si-NC) targeting hsa_circ_0005243 were transfected into the human trophoblast cell line HTR-8/SVneo. Interference efficiency was verified after transfection, with all three si-circRNAs constructs shown to significantly reduce the expression of hsa_circ_0005243 (Fig. 2a). Of these three constructs, si-circRNA-2 exhibited the most potent effect, so it was chosen for further experiments.

CCK8 analyses revealed a significant reduction in cell viability following si-circRNA treatment relative to the si-NC group at both 48 and 72 h after transfection (Fig. 2b). EdU staining revealed significantly fewer EdU-positive cells in the si-circRNA group compared to the control group (Fig. 2c). Similar results were shown in the clone formation experiment (Fig. 2d), with significantly fewer clones in the si-circRNA group compared to the si-NC control group. Flow cytometry analysis revealed a higher proportion of apoptotic cells after hsa_circ_0005243 knockdown (Fig. 2e).

### hsa_circ_0005243 knockdown inhibits migration of trophoblast cells

**Fig. 3:**
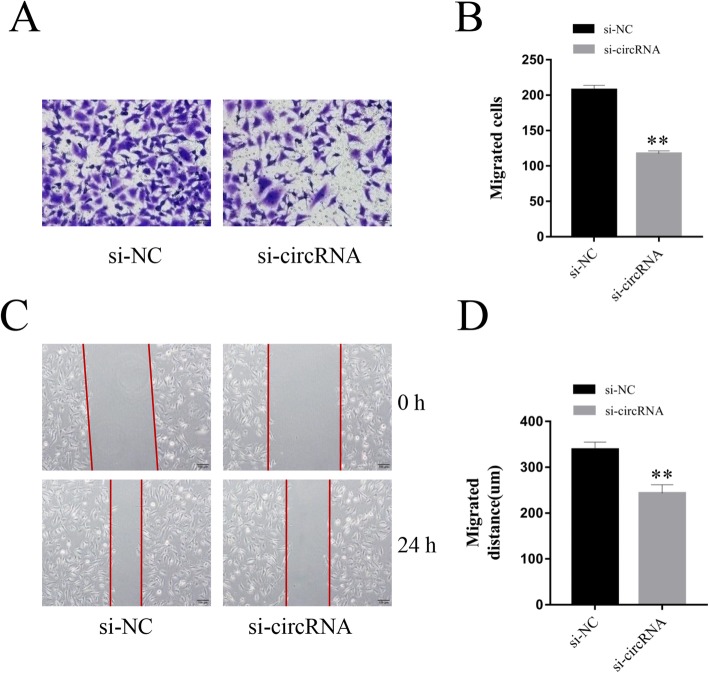
Knockdown of hsa_circ_0005243 inhibits cell migration. **a** An in vitro transwell assay showed a significant decrease in the migration ability of trophoblast cells following knockdown of hsa_circ_0005243, as evidenced by a decrease in migratory cell numbers. **b**. The number of migrated cells was counted for each group. **c** A wound-healing assay revealed a significant difference in migration distance before and after hsa_circ_0005243 knockdown. **d** The migratory distance was calculated 24 h after transfection. Data are presented as the mean ± SD, **p < 0.01

Normal migration of trophoblast cells is important for the maintenance of placental function [14]. Therefore, we investigated the effect of hsa_circ_0005243 on the migration ability of trophoblast cells. The transwell assay showed a significant decrease in the migration ability of trophoblast cells after transfection with si-circRNA (Fig. 3a), with fewer migratory cells in the knockdown group relative to controls (Fig. 3b). These observations were further supported by the results from the wound-healing assay, which showed a significantly shorter migration distance in the si-circRNA group compared with the control group (Fig. 3c and d).

### Knockdown of hsa_circ_0005243 elevates TNF-α and IL-6 levels

**Fig. 4:**
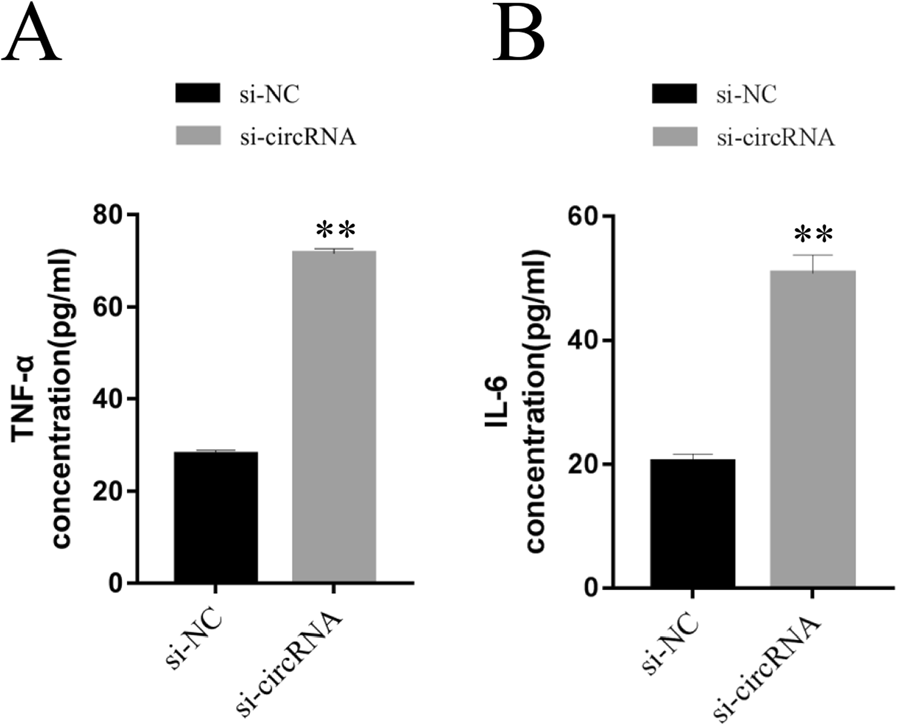
Knockdown of hsa_circ_0005243 promotes TNF-α and IL-6 expression. **a**, **b** Knockdown of hsa_circ_0005243 promoted TNF-α (**a**) and IL-6 (**b**) expression as determined by ELISA. Data are presented as the mean ± SD, **p < 0.01

Inflammation plays a significant role in GDM pathogenesis, with numerous inflammatory mediators regarded as risk factors for GDM development [15]. To assess the role of these factors in the context of hsa_circ_0005243, ELISA was used to detect inflammatory factor levels in culture medium. After hsa_circ_0005243 knockdown, the levels of TNF-α (Fig. 4a) and IL-6 (Fig. 4b) were significantly increased in the culture medium compared with the si-NC group.

### Potential regulatory mechanism of hsa_circ_0005243 in trophoblast cell function and inflammation

**Fig. 5:**
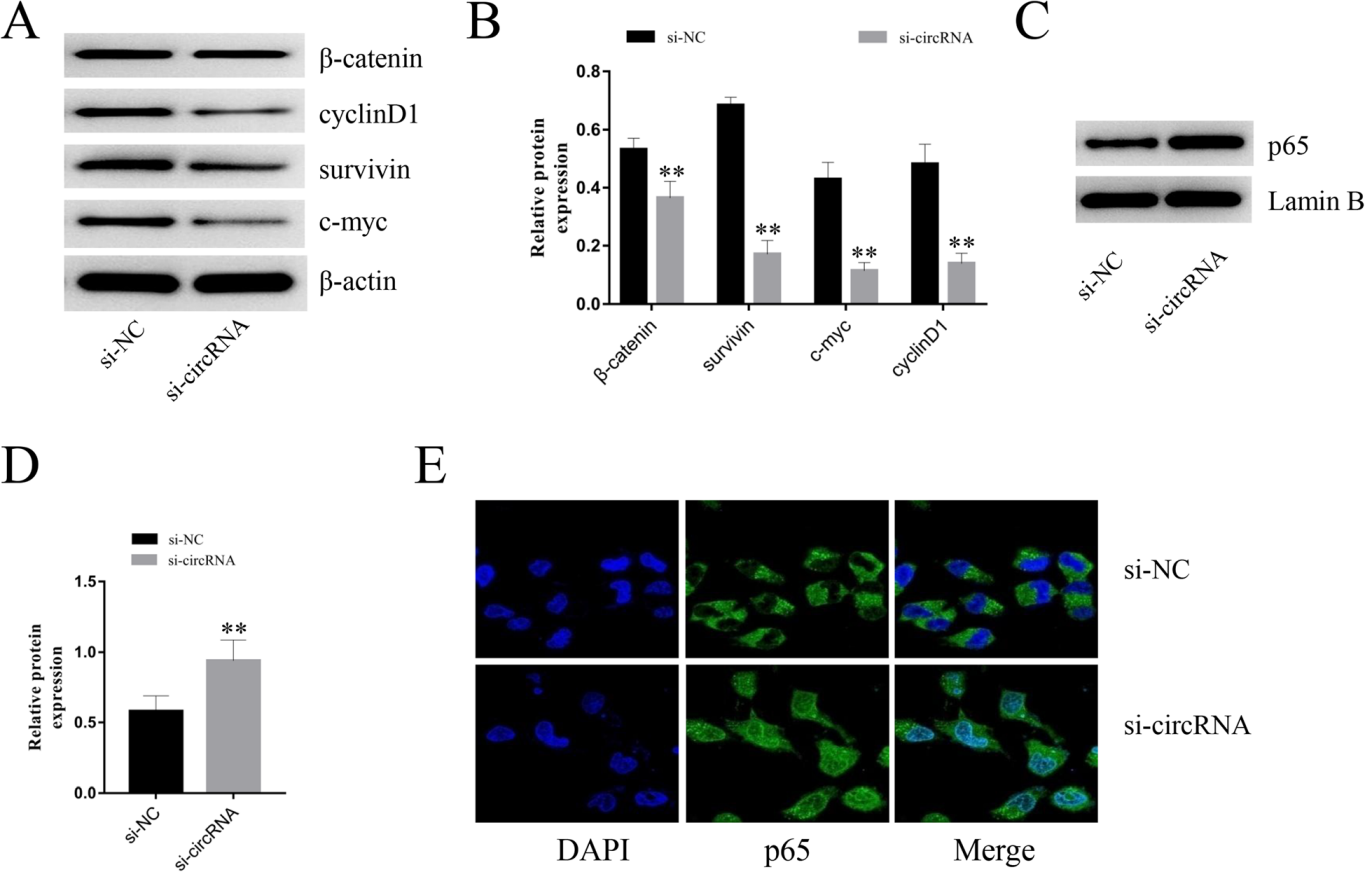
Potential mechanisms of hsa_circ_0005243 regulation of trophoblast cell function and inflammatory factor expression. **a** Following knockdown of hsa_circ_0005243 in HTR-8/SVneo cells, the expression of β-catenin, c-myc, cyclin D1, and survivin were measured by Western blot. **b** Quantitative analysis of protein expression. **c**, **d** The expression of p65 protein in the nucleus was elevated after hsa_circ_0005243 knockdown. **e** Confocal immunofluorescence analysis showed that depletion of hsa_circ_0005243 was associated with an increase in NF-kB p65 subunit nuclear translocation. Data are presented as the mean ± SD, **p < 0.01

To further investigate the potential molecular mechanisms underlying hsa_circ_0005243 activity in trophoblast cells, the expression of various signaling pathway proteins was detected by Western blot. β-catenin was significantly decreased after hsa_circ_0005243 knockdown, along with the expression of its related downstream molecules, including c-myc, cyclinD1, and survivin (Fig. 5a and b). In addition, nuclear NF-κB expression was increased after hsa_circ_0005243 depletion (Fig. 5c and d), with evidence of increased nuclear translocation of its p65 subunit via immunofluorescence assay (Fig. 5e).

## Discussion

Gestational diabetes is characterized by varying degrees of abnormal glucose metabolism during pregnancy, which can significantly affect both maternal and infant health. About 2–5% of all pregnant women develop GDM, with considerable increases in disease prevalence observed during the last decade [16]. GDM is associated with a wide range of serious complications, including dystocia, macrosomia, and neonatal hypoglycemia [2, 17]. Therefore, timely diagnosis and appropriate therapeutic intervention are important for reducing the risk of adverse pregnancy outcomes in GDM patients. Although the pathogenesis of GDM is not completely understood, recent studies have shown considerable overlap with the pathogenesis of type 2 diabetes mellitus, characterized by a synergistic effect of external environmental factors and permissive genetics. Pregnant women with a family history of type 2 diabetes mellitus had a significantly increased risk of GDM [18].

CircRNAs are a class of non-coding RNA molecules characterized by a closed circular structure. With the rise of high-throughput sequencing technologies, the number of reported circRNAs has risen dramatically in recent years, revealing important regulatory functions [19, 20] and indicating their potential value as both diagnostic and therapeutic targets for various diseases. From a mechanistic standpoint, circRNAs are thought to participate in gene regulation by acting as sponges for miRNAs, thereby limiting their inhibitory effects on their target genes [9]. Alternatively, circRNAs have been shown to target RNA-binding proteins as a mechanism of gene regulation [21], while other circRNAs have protein-coding functions of their own [22].

The placenta facilitates the transport of nutrients, gases, and other compounds between mother and fetus. A wide array of placental changes have been reported in patients with GDM [16, 23], including the differential expression of circRNAs [11, 13]. While preliminary studies have suggested a link between circRNA expression, disease pathogenesis, and pregnancy outcomes [24], the role of circRNAs in GDM remains poorly understood. In this study we found that the expression of hsa_circ_0005243 was significantly decreased in the placentas of patients with GDM. These observations were consistent with in vitro experiments that showed that knockdown of hsa_circ_0005243 suppressed cell proliferation and migration in HTR-8/SVneo trophoblast cells. Placental trophoblasts are among the most active cells in pregnancy, with dysfunction of these cells resulting in abnormal placental exchange between mother and fetus and increased inflammation, which may lead to adverse pregnancy outcomes. Although the pathogenesis of GDM is still unclear, chronic inflammation has been shown to play an important role [25]. We found the levels of the inflammatory factors TNF-α and IL-6 were increased after hsa_circ_0005243 knockdown. TNF-α is a cytokine secreted mainly by monocytic macrophages. During pregnancy, the placenta secretes TNF-α, which may result in increased aggregation and adhesion of inflammatory cells, and damage to the vascular endothelium. TNF-α plays an important role in glucose and lipid metabolism, and is closely related to insulin resistance and GDM; moreover, it is positively correlated with body mass index [26–28]. IL-6 is not only involved in the regulation of immune and inflammatory responses, but also plays an important role in the balance of energy metabolism. IL-6 is directly involved in the development of GDM, with its expression significantly increased in the placenta and plasma of GDM pregnant women [29, 30]. In this study, ELISA analyses revealed significantly higher TNF-α and IL-6 levels in the hsa_circ_0005243 knockdown group, further implicating this circRNA as an immune regulator.

To explore the potential mechanism of hsa_circ_0005243 activity, we examined a variety of signaling pathway molecules related to GDM, revealing significant decreases in the expression of β-catenin and its downstream targets. β-catenin is a component of the Wnt signaling pathway, which plays an important role in cell proliferation, apoptosis, migration, and invasion [31]. The WNT/β-catenin signaling pathway has been shown to play a role in trophoblastic stem cell differentiation, chorionic allantoic cell fusion, and placental morphological development in pregnant rats [32]. Downregulation of β-catenin was observed in the placental tissues of patients with preeclampsia and during hypoxia/reoxygenation of HTR-8/SVneo cells [33].

Changes in other genes associated with the Wnt canonical pathway have also been observed, including downregulation of Wnts, Fzds, β-catenin, Apc, and GSK-3β, suggesting regulation of Wnt expression by hyperglycemia in different embryonic tissues [34]. These findings suggest that maternal diabetes may suppresses Wnt signaling [35]. Therefore, we speculate that trophoblast dysfunction after hsa_circ_0005243 depletion may be involved in the pathogenesis of placental dysfunction. In addition, a decrease in nuclear NF-κB p65 expression was observed. The NF-κB pathway is a central regulator of the immune system, controlling stress responses, apoptosis, and inflammation. NF-κB belongs to the nuclear transcription factor family, existing as a dimer composed of p50 and p65 protein subunits. NF-κB p65 can interact with cytokines such as TNF-α and IL-6, and forms a positive feedback loop, thereby amplifying the inflammatory response in GDM [36–38]. Following activation by inflammatory factors such as TNF-α and IL-6, the p65 subunit enters the nucleus, where it drives transcription of a number of inflammatory factors, which further enhances inflammatory activity. This chronic immune activation can in turn drive insulin resistance, leading to the decrease of insulin sensitivity. Here, immunofluorescence assays revealed increased p65 protein accumulation in the nucleus after hsa_circ_0005243 knockdown.

Our in vitro cell experiment results showed that the decreased expression of hsa_circ_0005243 inhibited trophoblast proliferation. This phenomenon seems to contradict the larger placentas and fetal macrosomia that occur in GDM [39]. Recently, Peng et al. [40] reported decreased cell vitality and proliferation of HTR-8/SVneo cells under high glucose treatment. Such a situation may be the result of continuous high glucose toxicity [41], which is not contradictory to fetal overgrowth induced by glucose.

Over time, the chronic inflammatory state characteristic of GDM patients [42] leads to an increase in the number of placental macrophages, which further exacerbates the inflammatory response [5]. GDM placental morphology is characterized by a wide array of abnormalities, including increased placental volume and weight, fibroid necrosis, infarcts, immature villi differentiation, and capillary hyperplasia [39]. In this study, we found that downregulation of hsa_circ_0005243 suppressed the proliferation and migration of trophoblast cells, combined with increased expression of IL-6 and TNF-α. Based on these observations, we speculate that these attributes are associated with the long-term effects of inflammation on placental development. The structural and functional changes of the placenta in GDM patients are driven by a host of variables, including the quality of blood glucose control during the critical period of placenta development, the treatment mode, and the period and duration of metabolic disorder [43]. The placental villi that arise during early pregnancy exhibit significant self-protection mechanisms, protecting normal trophoblast function and inhibiting the process of inflammation [42], because failure or serious defects in placenta formation will lead to the loss of pregnancy. Therefore, successful pregnancy depends on the feedback, regulation, and adaptation of cytokines secreted by the maternal immune system and placenta [44]. Once the duration or degree of diabetes driven by maternal hyperglycemia, hyperinsulinemia, or dyslipidemia exceeds the placental regulatory capacity, fetal overgrowth will occur [43].

## Conclusions

In this study, we found that hsa_circ_0005243 was downregulated in GDM placenta. In vitro experiments verified that downregulation of hsa_circ_0005243 suppressed trophoblast proliferation and migration, while driving the production of the inflammatory cytokines IL-6 and TNF-α. Subsequent mechanistic studies showed that depletion of hsa_circ_0005243 significantly reduced the expression of β-catenin and its downstream targets. Furthermore, expression of NF-κB, which mediates the inflammatory response, was also increased, along with increased nuclear translocation of the p65 subunit. Although our research provides new potential molecular targets for the treatment of GDM, additional in vivo and in vitro studies are recommended due to the complexity of the regulatory mechanisms.

## Data Availability

All data generated or analysed in this study are included in this published article.

## Abbreviations

GDM: Gestational diabetes mellitus
qRT-PCR: quantitative reverse transcription polymerase chain reaction
circRNA: Circular RNA
NF-κB: Nuclear factor kappa-B
TMEM184B: Transmembrane protein 184B
